# Transient transcription factor (OSKM) expression is key towards clinical translation of *in vivo* cell reprogramming

**DOI:** 10.15252/emmm.201707650

**Published:** 2017-04-28

**Authors:** Irene de Lázaro, Giulio Cossu, Kostas Kostarelos

**Affiliations:** ^1^ Nanomedicine LabFaculty of Biology, Medicine and HealthThe University of ManchesterManchesterUK; ^2^ Division of Cell Matrix Biology & Regenerative MedicineFaculty of Biology, Medicine and HealthThe University of ManchesterManchesterUK

**Keywords:** Regenerative Medicine, Stem Cells

## Abstract

Reprogramming adult, fully differentiated cells to pluripotency *in vivo* via *Oct3/4*,* Sox2*,* Klf4* and *c‐Myc* (OSKM) overexpression has proved feasible in various independent studies and could be used to induce tissue regeneration owing to the proliferative capacity and differentiation potential of the reprogrammed cells. However, a number of these reports have described the generation of teratomas caused by sustained reprogramming, which precludes the therapeutic translation of this technology. A recent study by the Izpisúa‐Belmonte laboratory described a cyclic regime for short‐term OSKM expression *in vivo* that prevents complete reprogramming to the pluripotent state as well as tumorigenesis. We comment here on this and other studies that provide evidence that *in vivo* OSKM induction can enhance tissue regeneration, while avoiding the feared formation of teratomas. These results could inspire more research to explore the potential of *in vivo* reprogramming in regenerative medicine.

## *In vivo* cell reprogramming for tissue regeneration

Reprogramming specialized cells within an injured or degenerated tissue to a pluripotent‐like state has been proposed as a novel strategy to promote regeneration while bypassing limitations of traditional cell therapies, namely those associated with *ex vivo* cell manipulation, transplantation and engraftment. The rationale behind this approach is to take advantage of the proliferative capacity of the reprogrammed cells, and of their ability to re‐differentiate into mature phenotypes, to repopulate the damaged tissue (de Lazaro & Kostarelos, 2014). The use of a well‐defined combination of transcription factors that are able to induce pluripotency in a variety of tissues—the *Oct3/4*,* Sox2*,* Klf4* and *c‐Myc* (OSKM) cocktail (Takahashi & Yamanaka, 2006)—adds additional benefits of simplicity and versatility. Ultimately, *in vivo* reprogramming could provide new therapeutic avenues to treat a variety of conditions such as myocardial infarction, Parkinson's disease or stroke, to name a few, in which the loss of specific cell types cannot be addressed by currently established therapies.

**Figure 1:**
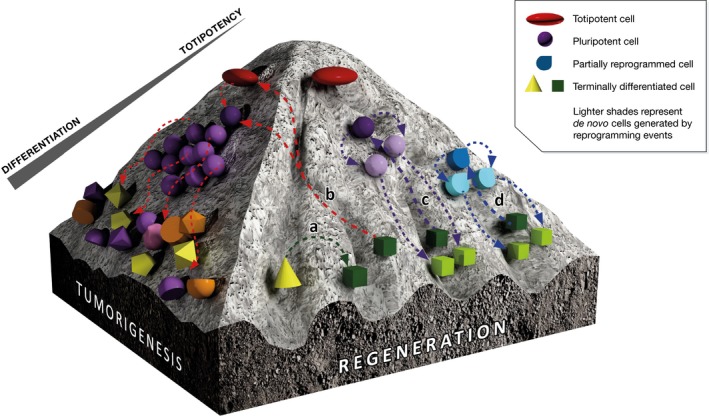
The fine line between tumorigenesis and regeneration upon *in vivo* reprogramming Various cell fate changes can be forced *in vivo*, albeit their outcomes differ. (a) Transdifferentiation drives direct conversion between specific cell types, but lacks the induction of cell division that maximizes the repopulation of an injured site; (b) sustained reprogramming to pluripotency leads to excessive and uncontrolled proliferation and random re‐differentiation into multiple lineages that results in the generation of teratomas; (c) *in vivo* reprogramming to a pluripotent or pluripotent‐like state may provide teratoma‐free tissue regeneration provided that the expression of pluripotency features and proliferation are strictly transient; (d) partial reprogramming is accompanied by transient proliferation that may replenish, among others, the pool of progenitor‐like cells crucial to maintain tissue homeostasis upon injury. This illustration has been adapted from the original idea of Waddington's epigenetic landscape.

*In vitro,* reprogramming is induced under defined conditions that promote and maintain pluripotency. However, an initial concern that casted doubts on the clinical translation of *in vivo* reprogramming is the fact that the pluripotent conversion within the organism has to overcome local pro‐differentiation signals that govern adult, fully differentiated tissues. While several independent studies have now demonstrated that the induction of pluripotency *in vivo* is indeed feasible via OSKM overexpression (Abad *et al*, 2013; Yilmazer *et al*, 2013; Ohnishi *et al*, 2014), the appearance of teratomas described by Abad *et al* (2013) and Ohnishi *et al* (2014) raised concerns about tumorigenesis and shifted the attention towards the safety of the approach. Both studies used a transgenic “reprogrammable” mouse—carrying an OSKM polycystronic cassette under the control of a doxycycline‐inducible promoter—to induce pluripotency within adult tissues. When such animals were fed with doxycycline for extended periods of time, ubiquitous and sustained expression of OSKM caused uncontrolled proliferation and disorganized differentiation followed by extensive tumorigenesis and death (Fig 1b) (Abad *et al*, 2013; Ohnishi *et al*, 2014). Both reports established a rather negative outlook for exploiting the therapeutic potential of *in vivo* reprogramming in regenerative medicine.

## Transient OSKM expression is key to prevent tumorigenesis and “rejuvenates” aged tissues

The same reports also suggested a direct relationship between the appearance of teratomas and the duration of OSKM expression. The doxycycline‐inducible promoter is in fact a convenient tool to control the *on* and *off* of OSKM expression with the administration and withdrawal of the drug; it allowed Abad *et al* (2013) to compare the effects of a high dose of doxycycline administered for a week to a five times lower dose over a longer interval. The shorter administration scheme resulted in lower tumorigenesis and mortality in spite of the higher dose (Abad *et al*, 2013). Ohnishi *et al* (2014) used four different induction schemes (doxycycline administration for 4, 5, 6 or 7 days) and also reported a direct relationship between the length of doxycycline feeding and the incidence of dysplastic lesions. Most of the animals fed with doxycycline for < 5 days did not show any histological aberration. Most importantly, short OSKM induction enabled re‐integration of at least some of the *in vivo* reprogrammed cells in the tissue. For example, after a transient de‐differentiated and proliferative phase, pancreatic cells re‐acquired their mature phenotype and physiological function and were able to produce insulin again (Ohnishi *et al*, 2014).

Now, a study by Izpisúa‐Belmonte and colleagues (Ocampo *et al*, 2016) has confirmed our earlier observations from the transient expression of OSKM in adult mouse liver (Yilmazer *et al*, 2013) and muscle (de Lazaro *et al*, 2017) that *in vivo* reprogramming can indeed completely escape tumorigenesis by limiting the duration of the expression of reprogramming factors. Moreover, Ocampo *et al* (2016) suggested that transient reprogramming erased various signs of ageing and enhanced the regenerative capacity of aged tissues. They proposed a partial reprogramming strategy in which epigenetic remodelling and active proliferation take place without reaching the pluripotent state (Fig 1d). Primary fibroblasts from progeroid reprogrammable mice—in which the onset of ageing starts at an aberrantly early age—were used to confirm that short‐term OSKM expression for 2–4 days was sufficient to trigger epigenetic changes that erased various hallmarks of the aged phenotype, but not enough to induce endogenous pluripotency markers, such as NANOG and SSEA1, and loss of cell identity. These cells were therefore “molecularly rejuvenated”, but not de‐differentiated to pluripotency.

However, maintaining the rejuvenated phenotype in progeroid cells required re‐induction of reprogramming factors, which prompted Ocampo *et al* (2016) to explore a short‐term but cyclic induction scheme to translate these findings to the *in vivo* set‐up. Progeroid reprogrammable mice were administered with doxycycline for 2 days, followed by a 5‐day withdrawal of the drug. The authors showed that six cycles of OSKM induction were sufficient to decrease various hallmarks of ageing, including histological alterations in a number of tissues and deficiencies in cardiac function, while dysplasia or teratomas were not observed for up to 35 cycles. Overall lifespan, which is severely compromised by the disease, was significantly increased in the OSKM group.

The study was also extended to physiological ageing, using 12‐month‐old, non‐progeroid mice. OSKM induction enhanced the otherwise poor regenerative capacity of both pancreas and skeletal muscle and made these tissues more resilient to a later insult. Cyclic induction of OSKM triggered proliferation of beta cells in the pancreas and satellite cells in the skeletal muscle, which are critical for the maintenance of tissue homeostasis, but whose numbers typically decrease with age. The expansion of these cell populations was thought to be the main cause to improved performance after streptozocin‐mediated ablation of beta cells (an accepted model of metabolic disease) and cardiotoxin‐induced muscle injury, a common experimental procedure to study muscle damage and regeneration. However, it would be interesting to interrogate this *in vivo* partial reprogramming approach in a sarcopenia model that better exemplifies the effects of physiological ageing in skeletal muscle.

## Transient *in vivo* reprogramming to repair injured tissues

The Ocampo *et al* (2016) study is the first to describe the potential of OSKM to improve the resilience of aged tissues to injury, but others have also proposed OSKM overexpression to induce or enhance cell replenishment and tissue repair after injury. Gao *et al* (2016) used retroviral vectors to specifically overexpress OSKM in glial cells, aiming to generate *de novo* cells to repair traumatic brain injury. However, although a number of reprogrammed cells re‐differentiated to the neural lineage, others expressed mesoderm and endoderm markers. More importantly, clusters of pluripotent‐like cells expressing NANOG and SSEA4 actively proliferated over time and eventually generated teratomas. These results confirm the limited clinical relevance of sustained *in vivo* reprogramming, in this case likely linked to the use of retroviral vectors.

With the specific goal of avoiding sustained reprogramming, our laboratory has used episomal plasmid DNA to induce OSKM *in vivo* (Yilmazer *et al*, 2013). In a recent report, we showed that the generation of cells within skeletal muscle tissue that proliferate and express pluripotency markers—including NANOG—only transiently, not only eludes tumorigenesis but also enhances regeneration in a clinically relevant skeletal muscle injury model that involved laceration of the medial gastrocnemius (Fig 1c) (de Lazaro *et al*, 2017).

Indeed, the existence of a transient proliferative phase upon reprogramming via OSKM induction, either as partial reprogramming (Fig 1d) or as a transient pluripotent‐like state (Fig 1c), may be more efficient in repopulating the injured site compared to transdifferentiation strategies (Fig 1a) that use cell‐specific transcription factors to mediate direct conversion between mature cell types, but lack the induction of cell division (Fig 1a). In addition, two studies have recently suggested that senescence signals, triggered by tissue damage and ageing, could favour and increase the efficiency of *in vivo* reprogramming (Chiche *et al*, 2016; Mosteiro *et al*, 2016). This interaction may reinforce the potential of the OSKM cocktail to assist regeneration of injured and aged tissues.

## Future directions and concluding remarks

Collectively, all these studies confirm that *in vivo* overexpression of OSKM can induce cell reprogramming without increasing the risk of teratoma formation. However, transient expression of such factors is key to avoid sustained pluripotency, uncontrolled proliferation and tumorigenesis, and should therefore be a prerequisite for clinical applications, either via partial *in vivo* reprogramming or through the generation of transient pluripotent‐like intermediate cells. The potential of the OSKM cocktail to enhance regeneration is encouraging in both strategies. To further explore therapeutic approaches, therefore, it is imperative that the field moves beyond any presumed association of *in vivo* reprogramming with teratoma formation and instead focuses on resolving the remaining challenges towards clinical translation.

Among those, the *in vivo* delivery of OSKM—or other reprogramming factors—will be a major issue and will greatly depend on the specific condition to be treated. In the context of a systemic disease, such as progeria, it may be unrealistic to raise expectations for a vector able to transduce all cells in a patient. Instead, it will be crucial to investigate whether reprogramming specific tissues, such as vital organs as the brain or liver, might be sufficient to halt or delay the life‐limiting progress of the disease. Even to reprogram a particular organ, tissue or cell type, engineering delivery vectors that provide targeted, efficient, transient and repeated expression of OSKM factors will be a crucial requirement to move from biologically interesting, yet clinically irrelevant, transgenic mouse models to clinical studies. In addition, as with any other therapeutic intervention, it will be important to account for potential species‐specific differences when aiming to identify the balance between efficient reprogramming and potential undesired effects. The history of clinical gene therapy offers many examples of strategies that worked very efficiently in animal models, but had detrimental outcomes in human patients (Wilson, 2009). Eventually, this will only be overcome with early‐stage, well‐designed and cautious human clinical studies.

In conclusion, we believe that the proof of concept of *in vivo* reprogramming for tissue regeneration and the strategies to elude tumorigenesis discussed here represent two initial prerequisites that should encourage further research towards clinical use. They will hopefully build enough confidence to continue on the long road to clinical translation of transient *in vivo* cell reprogramming for tissue regeneration and rejuvenation.

## Conflict of interest

The authors declare that they have no conflict of interest.
